# Health literacy among residents in communities implementing healthy community initiatives: a cross-sectional study

**DOI:** 10.3389/fpubh.2026.1860270

**Published:** 2026-06-23

**Authors:** Tingjie Liu, Dan Jiang, Ruijie Cai, Monan Xia, Haonan He, Zijia Cheng, Runtang Meng

**Affiliations:** Department of Preventive Medicine, School of Public Health and Nursing, Hangzhou Normal University, Hangzhou, China

**Keywords:** health, health literacy, health promotion, healthy community, influencing factors

## Abstract

**Objective:**

To examine the association between healthy community construction and residents’ health literacy and to provide evidence for optimizing health promotion strategies.

**Methods:**

A cross-sectional study was conducted using a multi-stage random sampling method. A total of 263 residents from two health-promoting communities in Xiaoshan District, Hangzhou, were surveyed, and the data were statistically analyzed.

**Results:**

The overall health literacy rate among residents was 65.78%, which was significantly higher than the reported local level in 2024 (53.18%) (*p* < 0.05). Higher literacy levels were observed in several dimensions, including healthy lifestyles and behaviors, basic health skills, health information, communicable disease prevention, and chronic disease prevention, compared with district-level reference data (*p* < 0.05). However, no significant improvement was found in basic knowledge and concepts (*p* > 0.05), and a decline was noted in literacy related to safety and first aid, scientific health concepts, and basic medical care. Logistic regression analysis indicated that being married (*OR* = 9.327), having chronic diseases (*OR* = 7.368), and preferring a meat-based diet (*OR* = 3.009) were positively associated with higher health literacy, whereas older age (*OR* = 0.321) and poorer self-rated health status (*OR* = 0.213) were negatively associated (*p* < 0.05).

**Conclusion:**

Residents from communities implementing Healthy Community initiatives showed relatively high levels of health literacy compared with district-level reference data. However, development across different dimensions remains uneven. Health education in areas such as scientific health concepts, safety and first aid, and basic medical care should be further strengthened. In addition, targeted interventions for older adults and other key populations are needed to promote sustained improvements in overall health literacy.

## Introduction

Health literacy refers to an individual’s capacity to obtain, understand, and apply basic health information and services in order to make appropriate health decisions, and it plays a critical role in preventing non-communicable diseases and mitigating the spread of misinformation (1). The World Health Organization (WHO) identifies health literacy, alongside life expectancy, maternal mortality, and infant mortality, as a key indicator for evaluating national public service provision and population health status. Substantial disparities in health literacy levels exist across countries. In China, the national health literacy rate was 31.87% in 2024 (2), In contrast, a survey of more than 8,000 residents across eight regions, including Australia, Germany, and Spain, found that only 16.5% of participants demonstrated excellent health literacy, while approximately 36.0% achieved sufficient levels (3). Limited health literacy undermines individuals’ ability to comprehend and utilize health information, leading to poor medication adherence, inadequate self-management, and insufficient engagement in preventive behaviors. Consequently, it increases the risk of chronic diseases and adverse health outcomes. At the societal level, low health literacy may result in inefficient use of healthcare resources, increased healthcare expenditures, and the widening of health inequalities (4–6).

To address these challenges, countries such as the United States, Australia, and Japan have implemented a range of health promotion and community-based health strategies (7–9). In response to rapid population aging, the increasing prevalence of lifestyle-related diseases, and rising healthcare expenditures, China launched the *Healthy China Action (2019–2030)* in 2019 through the National Health Commission. This initiative aims to achieve a national health literacy rate of at least 30% by 2030 and highlights the pivotal role of community-based health promotion in improving health literacy, preventing lifestyle-related diseases, and enhancing overall population health. These efforts may contribute to alleviating pressures associated with population aging and reducing the economic burden on the healthcare system (10).

In addition, evaluating the implementation outcomes of health promotion initiatives may provide important evidence base for improving health education, strengthening basic public health services, and optimizing health literacy promotion programs (11, 30). To date, numerous cross-sectional studies have investigated residents’ health literacy worldwide. For instance, Japan has evaluated the *Health Japan 21* initiative from the perspective of health management experts (12). Several studies in China have explored the relationship between health promotion initiatives and residents’ health literacy. For example, Gong et al. reported that the National Health Promotion County initiative was associated with improvements in residents’ health literacy levels (13), while Lin et al. analyzed demographic factors influencing health literacy among urban residents in Shenzhen (14). However, most existing studies primarily focused on overall literacy prevalence or influencing factors, whereas empirical evaluations of multidimensional differences in health literacy among communities implementing Healthy Community initiatives remain limited. This gap in the literature may hinder the precise optimization of such programs and potentially diminish the long-term effectiveness of health promotion efforts.

Against the backdrop of accelerated population aging, a growing burden of lifestyle-related diseases, and rising healthcare expenditures in China, achieving the target set in the *Healthy China Action (2019–2030)*—namely, attaining a national health literacy rate of at least 30% by 2030—has become increasingly urgent. Therefore, empirical research is needed to explore the association between health promotion initiatives and residents’ health literacy and to identify the associated influencing factors. Such efforts may help better understand the potential associations between community health initiatives and residents’ health behaviors and literacy, optimizing the utilization of healthcare resources, and promoting health equity.

This study focuses on community-level residents to comprehensively evaluate the real-world effectiveness of health promotion initiatives. It aligns with the strategic objectives of the *Healthy China* initiative and represents an important step toward improving health education and public health services, optimizing health promotion strategies, and alleviating the economic burden on the healthcare system. The findings are expected to provide both theoretical support for refining targeted health promotion interventions within national health initiatives and empirical evidence for enhancing residents’ health literacy and advancing population health and well-being in China.

## Materials and methods

### Study design

This study was designed as a cross-sectional survey conducted among residents from two communities in Xiaoshan District, Hangzhou, Zhejiang Province. All participants were provided with a study information sheet and an informed consent form prior to enrollment. Written informed consent was obtained from all participants after they had reviewed the study information. Following enrollment, questionnaires were administered to collect relevant data.

The study variables included demographic characteristics (age, ethnicity, sex, marital status, educational level, occupation, household registration status, presence of chronic diseases, smoking status, alcohol consumption, and dietary habits), health literacy, and self-rated health status. Health literacy was assessed across three dimensions: basic health knowledge and concepts, healthy lifestyles and behaviors, and basic health skills.

Demographic data were collected using self-administered questionnaires. Smoking status was categorized as daily, occasional, former, or never smoking. Alcohol consumption was classified as daily, 4–6 times per week, 1–3 times per week, less than once per month, or never. Dietary habits were assessed based on preferences for meat-based, salty, vegetarian, or light diets, with responses recorded as “yes” or “no.” Chronic disease status was also categorized as “yes” or “no.”

Health literacy was measured using the standardized Chinese Resident Health Literacy Questionnaire developed by the China Health Education Center in 2017 (15). The questionnaire evaluates three core dimensions: basic knowledge and concepts, healthy lifestyles and behaviors, and basic health skills, which are further subdivided into six domains. Item formats include single-choice questions, multiple-choice questions, and situational judgment questions. According to the official scoring criteria, correct responses receive one point, whereas incorrect or unanswered items receive zero points. Adequate health literacy was defined according to the official evaluation standards.

According to the evaluation criteria established by the China Health Education Center, a total score of ≥34 indicates adequate overall health literacy. For the three dimensions, scores of ≥15, ≥11, and ≥8 correspond to adequate literacy in basic knowledge and concepts, healthy lifestyles and behaviors, and basic health skills, respectively (15). Self-rated health status was assessed using a single-item 5-point Likert-type question, ranging from 1 (good) to 5 (poor).

Trained interviewers conducted face-to-face questionnaires in the selected communities. Before the formal survey, all interviewers received standardized training on questionnaire distribution and data quality control. Paper questionnaires were filled out anonymously on - site and collected immediately upon completion. Interviewers checked the completeness and logical consistency of all questionnaires to ensure data quality.

### Study participants

A stratified multi-stage sampling method combining probability proportional to size sampling (PPS) and random sampling was employed in this study. In June 2024, a cross-sectional survey was conducted among residents from communities in Xiaoshan District, Hangzhou, Zhejiang Province, where Healthy Community initiatives were being implemented. During the same period, routine district-wide monitoring of health literacy was also carried out. Considering the clustering of health literacy and health-related behaviors within households, only one eligible participant was selected from each household. Routine district-level health literacy surveillance in Xiaoshan District in 2024 adopted a multi-stage PPS sampling framework in which four subdistricts and corresponding communities were selected. Based on this surveillance framework, the present study specifically included residents from two health-promoting communities implementing Healthy Community initiatives as the final analytical sample. In each selected community, 80 households were randomly sampled, and trained investigators subsequently conducted face-to-face household interviews using paper-based questionnaires. One eligible resident aged 15–69 years was randomly selected from each household using the KISH method for participation in the survey until all target questionnaires for the community were completed. The inclusion criteria were residents aged 15–69 years who were permanent residents of the selected communities. The exclusion criteria included individuals aged <15 or >69 years and those who had resided in the community for less than 1 year. Among individuals who met the eligibility criteria (*n* = 268), a total of 263 valid responses were obtained, yielding an effective response rate of 98.1%. These 263 participants were included in the cross-sectional analysis.

### Analysis

Qualified questionnaires were coded and verified for accuracy. Descriptive analyses were performed using rates (%) to summarize health literacy levels (possession rates) across different dimensions among residents of Xiaoshan District in 2024 and within the study population. Demographic variables were appropriately coded, and differences in health literacy across demographic groups were assessed using Z-tests and chi-square tests. Binary logistic regression analysis was conducted to examine the associations between demographic factors and overall health literacy. To reduce potential bias caused by sparse data and small subgroup sizes, Firth penalized logistic regression was additionally performed as a sensitivity analysis. All statistical tests were two-sided, with a significance threshold of *p* < 0.05. Analyses were performed using IBM SPSS Statistics version 25.0 (Armonk, NY, USA) and R version 4.3.2 (R Foundation for Statistical Computing, Vienna, Austria).

### Ethics approval and consent to participate

This study was approved by the Institutional Review Board of Hangzhou Normal University (approval number: 2021–0014). All procedures were in accordance with the ethical standards of the responsible committee on human experimentation and with the Helsinki Declaration.

## Results

Table 1 presents the differences in health literacy levels between the study population and residents of Xiaoshan District, Hangzhou, in 2024. Routine district-level health literacy monitoring data from Xiaoshan District in 2024 were used as contextual reference data for descriptive comparison. Because the district-wide surveillance may have partially included residents from the investigated communities, the comparison should be interpreted cautiously and does not constitute an independent controlled comparison. No statistically significant difference was observed in the dimension of basic knowledge and concepts (*p* = 0.488), whereas all other dimensions and categories of health literacy demonstrated significant differences (*p* < 0.05). Except for safety and first aid, scientific health concepts, and basic medical literacy—where literacy levels were lower than the district average—the study population demonstrated relatively higher literacy levels across several dimensions compared with the district-level reference data.

**Table 1:** Comparison of residents’ health literacy levels after the implementation of healthy community initiatives.

Dimension/domain	Study participants (*n* = 263), %	Xiaoshan District Residents 2024, %	*Z*	*p*
Overall health literacy	65.78	53.18	4.10	<0.001
Basic knowledge and concepts	66.15	64.10	0.69	0.488
Healthy lifestyle and behaviors	63.88	49.79	4.57	<0.001
Basic health skills	66.16	45.77	6.64	<0.001
Safety and first aid literacy	70.72	78.66	−3.14	0.002
Scientific health beliefs literacy	68.82	74.17	−1.98	0.047
Health information literacy	71.48	60.05	3.78	<0.001
Communicable disease prevention literacy	58.17	38.51	6.55	<0.001
Chronic disease prevention literacy	67.68	52.01	5.09	<0.001
Basic medical literacy	12.55	36.23	−7.99	<0.001

*p < 0.05 indicate statistically significant differences between study participants and district-level residents.

Table 2 summarizes the demographic characteristics of the study participants and the results of univariate analyses. Among the 263 participants, the mean age was 35.71 ± 15.03 years, with 45.6% aged 15–30 years. The sample comprised 165 males (62.7%) and 98 females (37.3%), and 67.7% were married. The study population was highly educated, with 73.4% holding a college degree or higher, compared with 3.8% having completed primary school or less. Occupationally, 35.4% were employed in enterprises, 10.3% were workers, and 4.9% were civil servants. Local household registration accounted for 72.6, and 13.7% of participants reported having chronic diseases.

**Table 2:** Univariate analysis of factors associated with health literacy among residents from healthy community initiative areas.

Variable		Surveyed (n)	Adequate health literacy (n)	Prevalence (%)	$\chi^{2}$ */Z*	*p*
Age (years)	15~	120	86	71.7	18.82	<0.001
	31~	102	69	67.6		
	41~	30	17	56.7		
	51 ~ 69	11	1	9.1		
Ethnicity	Han	255	172	67.5	8.11	0.004
	Minority	8	1	12.5		
Sex	Male	165	122	73.9	12.14	<0.001
	Female	98	51	52.0		
Marital status	Married	178	129	72.5	10.06	0.002
	Unmarried	85	44	51.8		
Education level	≤Primary	10	1	10.0	33.00	<0.001
	Junior school	14	6	42.9		
	High school	46	22	47.8		
	College/bachelor	172	125	72.7		
	Master and above	21	19	90.5		
Occupation	Civil servant	13	9	69.2	31.05	<0.001
	Teacher	10	7	70.0		
	Healthcare worker	11	9	81.8		
	Public institution	12	10	83.3		
	Student	9	7	77.8		
	Farmer	8	0	0		
	Worker	27	20	74.1		
	Enterprise staff	93	70	75.3		
	Other	80	41	51.2		
Registration	Local	191	149	78.0	44.40	<0.001
	Non-local	72	24	33.3		
Chronic disease	Yes	36	2	5.6	64.14	<0.001
	No	227	171	75.3		
Smoke	Daily	18	6	33.3	24.73	<0.001
	Occasional	25	15	60.0		
	Former	18	5	27.8		
	Never	202	147	72.8		
Alcohol consumption	Daily	8	1	12.5	31.02	<0.001
	4–6 times/week	7	2	28.6		
	1–3 times/week	14	7	50.0		
	1–3 times/month	29	12	41.4		
	<1 time/month	33	21	63.6		
	Never	172	130	75.6		
Diet—meat-based	Yes	119	98	82.4	25.19	<0.001
	No	144	75	52.1		
Diet—vegetarian	Yes	62	44	71.0	0.69	0.405
	No	201	129	64.2		
Diet—salty	Yes	147	109	74.1	9.55	0.002
	No	116	64	55.2		
Diet—light	Yes	88	51	58.0	3.09	0.079
	No	175	122	69.7		
Self-rated health	Good	171	150	87.7	107.41	<0.001
	Relatively good	55	17	30.9		
	Fair	28	5	17.9		
	Relatively poor	4	1	25.0		
	Poor	5	0	0		

*Prevalence (%) = (Adequate Health Literacy n/Surveyed n) × 100. Variables with *p* < 0.05 indicate a statistically significant association with health literacy.

The prevalence of smoking and alcohol consumption was 16.3 and 34.6%, respectively, while 45.2% reported engaging in regular physical activity. Dietary habits included light (33.5%), salty (55.9%), meat-based (45.2%), and vegetarian (23.6%) diets. Fifty-five participants (20.9%) rated their health as relatively good. Univariate analyses indicated that age, ethnicity, sex, marital status, education level, occupation, household registration, chronic disease status, smoking, alcohol consumption, physical activity, meat-based and salty diets, and self-rated health were all significantly associated with overall health literacy (*p* < 0.05).

Binary logistic regression was performed with overall health literacy after healthy community construction (0 = not adequate, 1 = adequate) as the dependent variable, and all variables found to be significant in the univariate analysis as independent variables. The model demonstrated good fit (*R*^2^ = 72.2%, *F* = 189.417, *p* < 0.001). Variable coding is presented in Table 3. The results indicated that being married, without chronic diseases, and a preference for a meat-based diet were positively associated with adequate health literacy, whereas older age and poorer self-rated health were negatively associated (*p* < 0.05). Detailed findings are provided in Table 4. Otherwise, the results of Firth penalized logistic regression were generally consistent with those of the conventional logistic regression model, indicating that sparse-data bias did not substantially affect the main findings.

**Table 3:** Coding of independent variables for logistic regression analysis.

Variable	Coding
Age (years)	15 ~ 30 = 1; 31 ~ 40 = 2; 41 ~ 50 = 3; 51 ~ 69 = 4
Marital status	Unmarried = 0; Married = 1
Chronic disease	No = 0; Yes = 1
Diet—meat-based	No = 0; Yes = 1
Self-rated health	Good = 1; Relatively good = 2; Fair = 3; Relatively poor = 4; Poor = 5

**Table 4:** Logistic regression analysis of factors associated with adequate health literacy among residents from healthy community initiative areas.

Variable	*B*	SE	Wald $\;\chi^{2}$	*p*	OR	95% CI
Age (years)	−1.137	0.339	11.236	0.001	0.321	0.165 ~ 0.624
Marital status	2.220	0.612	13.153	<0.001	9.207	2.774 ~ 30.558
Chronic disease	−1.997	0.893	5.005	0.025	0.136	0.024 ~ 0.781
Diet—meat-based	1.102	0.488	5.093	0.024	3.009	1.156 ~ 7.832
Self-rated health	−1.547	0.345	20.070	<0.001	0.213	0.108 ~ 0.419
Constant	−2.504	1.915	1.710	0.191	0.082	

*B, regression coefficient; SE, standard error of B; Wald *χ*^2^, Wald test statistic; OR, odds ratio; CI, confidence interval. The model fit: *F* = 189.417, *R*^2^ = 0.722, *p* < 0.001.

## Discussion

According to the Xiaoshan District Health Commission, the overall prevalence of adequate health literacy among residents in 2024 was 53.18%. In the present survey, the 263 participants exhibited an overall health literacy rate of 65.78%, which was higher than the reported district-level reference value (*p* < 0.05). Under the framework of the *Healthy China Action (2019–2030)*, Xiaoshan District launched Healthy Community initiatives in 2019. Priority projects were tailored to the specific characteristics of each village and community, including hypertension prevention and the development of older adult(s) friendly living environments, supported by annual intervention plans.

Efforts were implemented across multiple dimensions—promoting healthy lifestyles, optimizing health services, strengthening health security, creating supportive environments, advancing health governance, and fostering healthy families—to promote public health awareness and support local health management. The establishment of health-promoting environments may be associated with greater public attention to health awareness and healthy lifestyle behaviors (16, 17). Residents from the investigated communities demonstrated relatively higher levels of overall health literacy and several health literacy dimensions compared with the district-level reference data: in the dimensions of healthy lifestyles and behaviors and basic health skills, and in the domains of health information, communicable disease prevention, and chronic disease prevention, compared with district-level reference data (*p* < 0.05). Although the literacy level for basic knowledge and concepts was relatively higher, the change was not statistically significant (*p* > 0.05), indicating the need for continued emphasis in this area. In contrast, literacy levels related to safety and first aid, scientific health beliefs, and basic medical literacy were lower than the district-level reference data. Particularly in the context of major public health emergencies, ensuring residents have adequate basic medical support, fostering scientific health beliefs, and providing fundamental knowledge on safety and first aid are critical. These findings underscore key areas for future focus in Healthy Community initiatives.

Multiple studies have reported a negative association between age and health literacy (18–20), consistent with our finding that residents aged 15–30 years exhibited higher health literacy levels following the Healthy Community initiative. Younger individuals generally have a greater capacity to acquire new knowledge and concepts and demonstrate better adherence in obtaining health information and modifying unhealthy lifestyle behaviors (15). In contrast, older adults often have lower educational attainment and, combined with age-related physiological decline, encounter greater challenges in accessing, understanding, and applying health knowledge to adopt healthier lifestyle practices (21).

Research by Zhang et al. (22) indicates that marital relationships provide stable emotional and informational support, thereby enhancing individuals’ ability to obtain and comprehend health information. Consistent with this, our findings demonstrate that married participants exhibited higher health literacy following the health promotion program. Married individuals may be more likely to maintain regular lifestyle patterns and engage with healthcare services healthcare services and health education resources, which contributes to improved health literacy for both themselves and their spouses (23).

The survey revealed that residents with chronic diseases exhibited lower health literacy, which is generally consistent with previous studies showing that chronic disease burden is associated with difficulties in accessing, understanding, and applying health information (24). Individuals with chronic conditions often face more complex healthcare needs and greater challenges in disease self-management, which may negatively affect their overall health literacy. In addition, age-related decline, lower educational attainment, and long-term disease burden may further limit their ability to obtain and utilize health information effectively (25).

In addition, both prior studies and the present research observed that individuals with a preference for a meat-based diet tend to exhibit higher health literacy. Generally, individuals with greater health literacy maintain more diverse and balanced dietary patterns, including moderate consumption of animal-based foods (26). In the context of China’s ongoing nutritional transition, this association may also be influenced by socioeconomic factors and dietary diversity. Specifically, socioeconomic status is positively correlated with health literacy (27, 28); individuals with higher socioeconomic status often consume more varied diets and concurrently demonstrate higher levels of health literacy.

Self-rated health status, as a subjective assessment of one’s own health, is often influenced by objective health conditions (29). Our findings indicate a significant positive association between self-rated health and health literacy, consistent with previous studies. Individuals with higher health literacy are generally better able to access and comprehend health information, adopt proactive health behaviors, and utilize healthcare services effectively. These behaviors are generally associated with better objective health outcomes and more positive subjective health perception. In turn, positive self-assessments of health reinforce healthy behaviors, fostering further improvements in health literacy and establishing a beneficial feedback loop (12).

This study provides a cross-sectional assessment of residents’ health literacy in communities implementing Healthy Community initiatives in Xiaoshan District, offering substantial practical relevance and policy guidance. Unlike previous studies that primarily reported overall health literacy levels, our study conducted a detailed, multidimensional analysis across healthy lifestyles and behaviors, basic health skills, and health knowledge, revealing variations across different health literacy dimensions and enabling descriptive comparison with district-level reference data. Furthermore, this study examined multiple factors including age, marital status, chronic disease status, dietary patterns, and self-rated health to explore the mechanisms underlying disparities in health literacy from both individual and social perspectives, thereby enhancing understanding of its determinants. In the context of rapid population aging and a rising burden of chronic diseases, systematically evaluating Healthy Community initiatives and their potential associations with health literacy is important for optimizing health promotion strategies, strengthening primary-level public health services, and advancing health governance.

Although this study provides valuable insights into the effectiveness of Healthy Community initiatives, several limitations should be acknowledged. First, the study employed a cross-sectional design, which precludes the establishment of temporal or causal relationships between Healthy Community initiatives and residents’ health literacy levels. In addition, because baseline data prior to the implementation of the initiatives were unavailable, the present study could not evaluate longitudinal changes in health literacy over time. Second, some regression estimates exhibited relatively wide confidence intervals, particularly for marital status and chronic disease status, suggesting potential instability of the model due to limited subgroup sample sizes, furthermore, relatively small sample size may limit the representativeness and generalizability of the findings. Third, health literacy was primarily assessed using self-administered questionnaires, which may be subject to information bias, particularly for self-rated health and behavior-related indicators. Otherwise, because detailed demographic distributions of the district-level surveillance population were unavailable, we could not perform age-standardized or multivariable-adjusted comparisons between the study sample and district-level residents. Therefore, differences in health literacy levels should be interpreted cautiously, as they may partially reflect demographic and socioeconomic differences rather than community initiative implementation alone. Future research should expand the sample size, adopt longitudinal or interventional study designs, and incorporate objective measures to enhance the scientific rigor and reliability of findings.

## Conclusion

This study demonstrated that the overall prevalence of adequate health literacy among residents in Xiaoshan District, Hangzhou, found that residents from communities implementing Healthy Community initiatives exhibited relatively high levels of health literacy compared with district-level reference data; development across different health literacy dimensions remains uneven. However, causal relationships cannot be established due to the cross-sectional design. The attainment of adequate health literacy was influenced by factors such as age, marital status, chronic disease status, dietary patterns, and self-rated health. Accordingly, targeted and personalized health promotion strategies are recommended for key populations, including older and unmarried residents. Overall, The findings may provide preliminary evidence supporting the potential role of Healthy Community initiatives in health literacy promotion, further optimization of interventions is needed to address weaker domains and priority populations, thereby enhancing overall health literacy and strengthening primary-level health governance.

## Data Availability

The original contributions presented in the study are included in the article/Supplementary material, further inquiries can be directed to the corresponding author.
