# Supplementary material for: Health literacy among residents in communities implementing healthy community initiatives: a cross-sectional study
Source: Front Public Health. 2026 Jun 23;14:1860270. doi: 10.3389/fpubh.2026.1860270 (PMC13337897; doi:10.3389/fpubh.2026.1860270)
Supplement: Supplementary file 1 [file Data_Sheet_1.DOCX]

**Residents’ Health Promotion Initiative Survey Questionnaire**

Dear Resident,

Hello!

To better understand residents’ health literacy, lifestyle behaviors, and awareness of health promotion initiatives, we are conducting this anonymous survey. The purpose of this questionnaire is to collect information for scientific research and health service improvement. Your participation is voluntary, and all information provided will be kept strictly confidential and used for research purposes only.

This questionnaire was adapted from the *Chinese Resident Health Literacy Questionnaire* developed by the China Health Education Center in 2017.

There are no right or wrong answers. Please answer each question honestly according to your actual situation and personal understanding. Unless otherwise specified, please select the most appropriate option. For multiple-choice questions, more than one option may be selected.

Thank you very much for your support and cooperation!

**I. True or False Questions**

*(Please select the option you think is correct.)*

**A01.**The best way to prevent influenza is to take antibiotics (anti-inflammatory drugs).

○ True

○ False

**A02.**

Intravenous infusion (IV drip) works better and faster, so it should be the first choice after getting sick.

○ True

○ False

**A03.** Fruits and vegetables have similar nutritional components, so eating fruits can replace eating vegetables.

○ True

○ False

**A04.** Children and adolescents may also develop depression.

○ True

○ False

**A05.** “Long illness makes a good doctor”; patients with chronic diseases can adjust their treatment plans according to their own feelings.

○ True

○ False

**A06.** Problems and diseases detected during health examinations do not require immediate action if there are no symptoms.

○ True

○ False

**II. Single-Choice Questions**

*(Only one option is correct for each question.)*

**B01.** Which of the following best describes the concept of health?

○ Health means being physically strong and free from disease

○ Health means having good mental quality and physical strength

○ Health is not only the absence of disease, but also a state of complete physical, mental, and social well-being

○ Don’t know

**B02.** Which of the following are possible routes of hepatitis B transmission?

○ Working, eating, or swimming together with infected individuals

○ Sexual contact, blood transfusion, and mother-to-child transmission

○ Talking, shaking hands, or hugging infected individuals

○ Don’t know

**B03.** Which statement about self-monitoring blood pressure is incorrect?

○ Self-monitoring blood pressure is useful for diagnosing hypertension

○ Regular self-monitoring helps doctors evaluate treatment effectiveness

○ Hypertensive patients do not need regular follow-up visits if their self-monitored blood pressure is stable

○ Don’t know

**B04.** Which of the following is NOT an early warning sign of cancer?

○ Abnormal lumps in the body

○ Blood in stool without obvious cause

○ Weight gain

○ Don’t know

**B05.** What should rescuers do first when someone has carbon monoxide poisoning?

○ Give the patient water

○ Move the patient to a well-ventilated area

○ Call emergency services and send the patient to hospital

○ Don’t know

**B06.** Which statement about tuberculosis treatment is correct?

○ There are no preferential policies

○ The government provides free anti-tuberculosis drugs

○ Hospitalization is free

○ Don’t know

**B07.** The most important harm caused by iodine deficiency is:

○ SARS infection

○ Impaired intelligence and growth development

○ Hypertension

○ Don’t know

**B08.** During vigorous exercise, people lose water through sweating. In this case, the best drink is:

○ Plain boiled water

○ Sugary beverages

○ Light salt water

○ Don’t know

**B09.** Which statement about National Basic Public Health Services is incorrect?

○ Mainly carried out in large hospitals

○ Carried out in primary healthcare institutions

○ Available free of charge to residents

○ Don’t know

**B10.** Under which circumstance should vaccination for children be postponed?

○ During a cold or fever

○ When crying

○ Within half an hour after eating

○ Don’t know

**B11.** What is the correct action when fever occurs?

○ Seek medical attention promptly

○ Take fever medicine based on past experience

○ Wait and observe first

○ Don’t know

**B12.** If adverse reactions occur after taking medicine according to a doctor’s prescription, what should the patient do?

○ Stop taking the medicine on their own

○ Consult a doctor

○ Continue taking the medicine

○ Don’t know

**B13.** If a medicine label contains the mark “OTC”, it means the medicine is:

○ Prescription medicine requiring a doctor’s prescription

○ Over-the-counter medicine that can be purchased without a prescription

○ Health supplement

○ Don’t know

**B14.** During influenza season, windows should be opened frequently for ventilation. Which statement is incorrect?

○ Windows should rarely or never be opened in winter to avoid catching colds

○ Ventilation dilutes bacteria and viruses indoors

○ Ventilation allows sunlight indoors, helping kill bacteria and viruses

○ Don’t know

**B15.** What is the correct way to read a glass thermometer?

○ Hold the mercury end horizontally when reading

○ Hold the glass end vertically when reading

○ Hold the glass end horizontally when reading

○ Don’t know

**B16.** If mild skin burns develop blisters, what is the correct action?

○ Break the blisters for faster recovery

○ Leave small blisters intact but break large blisters

○ Do not break blisters to avoid infection

○ Don’t know

**B17.** What is the correct way to escape during a fire?

○ Protect the head with hands or clothes and rush out

○ Pour water over the body or wrap the body in a wet blanket before escaping

○ Beat flames with clothes while escaping

○ Don’t know

**B18.** Fever is a common symptom of Influenza. At what body temperature should a person seek medical attention?

○ 37.0°C

○ 37.5°C

○ 37.3°C

○ Don’t know

**III. Multiple-Choice Questions**

*(Two or more options may be correct.)*

**C01.** Which of the following methods help promote mental health?

□ Maintain an optimistic attitude toward life

□ Set goals within one’s abilities

□ Build good interpersonal relationships and actively participate in social activities

□ Relieve stress through smoking or drinking alcohol

□ Don’t know

**C02.** Which of the following statements are correct?

□ It is too late for elderly people to treat osteoporosis

□ Osteoporosis is a normal physiological phenomenon of aging

□ Drinking milk can reduce bone loss in middle-aged and elderly people

□ Exercise can help prevent osteoporosis

□ Don’t know

**C03.** What measures should be taken for a person with respiratory and cardiac arrest?

□ Artificial respiration

□ Chest compressions

□ Call emergency services

□ Give antihypertensive medication

□ Don’t know

**C04.** Benefits of eating soybean products such as tofu and soy milk include:

□ Beneficial to overall health

□ Beneficial for cardiovascular disease patients

□ Increase intake of high-quality protein

□ Reduce negative effects of excessive meat consumption

□ Don’t know

**C05.** Benefits of exercise include:

□ Maintaining an appropriate body weight

□ Preventing chronic diseases

□ Reducing psychological stress

□ Improving sleep

□ Don’t know

**C06.** When coughing or sneezing, the correct actions are:

□ Cover the mouth and nose directly with hands

□ Cover the mouth and nose with a tissue or handkerchief

□ Cover the mouth and nose with the elbow

□ Do not cover the mouth or nose

□ Don’t know

**C07.** During thunderstorms outdoors, which actions are correct?

□ Shelter under a large tree

□ Stay away from power lines

□ Avoid using mobile phones

□ Stand in high places

□ Don’t know

**C08.** Who can serve as sources of Influenza infection?

□ Influenza patients

□ Asymptomatic carriers of Influenza

□ Family members of Influenza patients

□ Doctors treating Influenza patients

□ Don’t know

**C09.** Through which routes can Influenza spread between people?

□ Sneezing

□ Coughing

□ Speaking loudly

□ Mosquito bites

□ Shared elevator buttons or door handles

□ Don’t know

**C10.** During the Influenza pandemic, which protective measures should individuals take?

□ Wear masks outdoors

□ Wash hands frequently

□ Ensure indoor ventilation

□ Avoid gatherings and group meals

□ Avoid crowded places

□ Take preventive medicine

□ Cover mouth and nose when coughing or sneezing

□ Maintain at least 1 meter of social distance

□ Don’t know

**IV. Scenario Questions**

**D01.** Mr. Li is 45 years old, 170 cm tall, and weighs 160 jin (80 kg). How should his BMI be calculated?

○ (80)^2^ / 170 = 37.6

○ 80 / (1.7)^2^ = 27.7

○ 160 / (1.7)^2^ = 55.4

○ Don’t know

**D02.** Which of the following methods can Mr. Li use to control his weight? *(Multiple-choice)*

□ Avoid staple foods completely

□ Exercise at least 30 minutes daily

□ Reduce fat intake

□ Eat only fruits and vegetables

□ Don’t know

**D03.** Which disease is Mr. Li more likely to develop?

○ Hypertension

○ Osteoporosis

○ Gastric ulcer

○ Don’t know

**V. Lifestyle**

**E01.** Do you smoke?

○ Smoke every day

○ Smoke occasionally

○ Have quit smoking

○ Never smoke

**E02.** Do you drink alcohol?

○ Drink every day

○ Drink 4–6 days per week

○ Drink 1–3 days per week

○ Drink 1–3 days per month

○ Drink less than once per month

○ Do not drink

**E03.** What is your dietary pattern? *(Multiple-choice)*

□ Prefer salty foods

□ Prefer light foods

□ Prefer meat-based foods

□ Prefer vegetarian foods

**VI. Basic Information**

**F01.** Gender:

○ Male

○ Female

**F02.** Date of Birth: ____ Year ____ Month

**F03.** Ethnicity:

○ Han

○ Minority

**F04.** Marital Status:

○ Married

○ Unmarried

**F05.** Education Level:

○ Primary school and below

○ Junior school

○ High school

○ College / Bachelor

○ Master and above

**F06.** Occupation:

○ Civil servant

○ Teacher

○ Healthcare worker

○ Public institution staff

○ Student

○ Farmer

○ Worker

○ Enterprise staff

○ Other: __________

**F07.** Are you a local registered resident?

○ Yes

○ No

**F08.** Do you currently have any chronic diseases (e.g., Hypertension, Diabetes)?

○ Yes

○ No

**F09.** Over the past year, how would you rate your overall health (Self-rated Health)?

○ Good

○ Relatively good

○ Fair

○ Relatively poor

○ Poor

**The survey is now complete. Thank you again for your support and cooperation!**
